# GPi/GPe borderland– a potential sweet spot for deep brain stimulation for chorea in Huntington’s disease?

**DOI:** 10.1186/s42466-024-00316-5

**Published:** 2024-05-23

**Authors:** Julia Steinhardt, Simone Zittel, Vera Tadic, Volker Tronnier, Christian Moll, Tobias Bäumer, Alexander Münchau, Dirk Rasche, Norbert Brüggemann

**Affiliations:** 1 Department of Neurology and Institute of Neurogenetics, University of Lübeck, Ratzeburger Allee 160, 23538 Lübeck, Germany; 2 Center of Brain, Behavior, and Metabolism, University of Lübeck, Lübeck, Germany; 3 Department of Neurology, University Medical Center Hamburg-Eppendorf, Hamburg, Germany; 4 Institute of Neurogenetics, University of Lübeck, Lübeck, Germany; 5 Department of Neurosurgery, University of Lübeck, Lübeck, Germany; 6 Institute of Systems Motor Sciences, University of Lübeck, Lübeck, Germany

**Keywords:** Huntington’s disease, Deep brain stimulation, Chorea, Volume of tissue activated, Globus pallidus externus

## Abstract

**Background:**

Pallidal deep brain stimulation (GPi-DBS) has been considered as an effective treatment option for medication-refractory Huntington’s disease (HD).

**Objectives:**

To identify stimulation-dependent effects on motor symptoms and to determine if these alterations are associated with the local impact of DBS on different pallidal parcellations.

**Methods:**

We prospectively evaluated the effects of bilateral GPi-DBS within one year in 5 HD patients. We evaluated the effects of GPi-DBS on choreatic symptoms and UHDRS. Electrode placement in the pallidum was localized, and the local impact of DBS was estimated.

**Results:**

The chorea subscore (*p* < 0.001) and UHDRS total motor score was significantly reduced postoperatively (*p* = 0.019). Pallidal DBS did not improve other motor symptoms. Activation of the lateral GPi/GPe was associated with improvement in choreatic symptoms (*p* = 0.048; *r* = 0.90).

**Conclusions:**

Our findings indicate that stimulation of the lateral GPi has a stable effect on choreatic symptoms. The modulation of the electrical field is relevant for motor outcome.

Deep brain stimulation (DBS) of the globus pallidus internus (GPi) is considered a rescue therapy for medically refractory chorea in Huntington’s disease (HD) [1–3]. A recent study evaluated clinical effects of GPi-DBS 6 and 12 months postoperatively showing a beneficial effect on chorea but not on HD-related parkinsonism and dystonia [1]. However, the application of DBS in HD remains challenging due to the low number of published cases, their heterogeneous phenotypic presentation, and the varying degree of brain atrophy [1]. Also, the optimal target has not yet been validated for HD. A first step to optimize electrode placement could be to identify the subregion in the pallidal area that is associated with the best outcome in patients that have already been operated.

Here, we re-assessed imaging data from 5 of 6 previously published patients with HD and pallidal DBS to identify a stimulation ‘hotspot’ that may be associated with better postoperative outcomes. All patients underwent bilateral DBS implantation with either the electrode model 3387 (Medtronic) or pendant electrodes (Libra XP, St. Judes Medical, Abbott). We used a state-of-the-art method for reconstruction of the electrode location and volume of tissue activated (VTA) calculation using LEAD DBS toolbox version 2.3.5 within MATLAB 2019 (The MathWorks, USA) with pre- and post-operative MRIs obtained at 1.5T or post-operative CT scans and individual stimulation parameters [4]. VTA, as an approximation of the DBS-activated tissue, was modeled using the TOR-PSM Atlas [5]. All clinical assessments were performed in a blinded manner and correlations with VTA were calculated for GPe, GPi, dystonia-GPi-coldspot, and dystonia-GPi-hotspot as given in the TOR-PSM atlas. Only significant correlations are reported. Based on our previous findings, we restricted our analysis to the chorea subscore of the Unified Huntington’s Disease Rating Scale. Data were given as mean ± SD and entered into a repeated measures analysis of variance. Posthoc t-tests were performed with a significance level of *p* < 0.05. Spearman rank correlation was used for clinical data and VTA, to test for an association. Although we used formal statistical tests, the analysis was only exploratory due the low number of cases.

In line with our previous publication [1], chorea improved in this slightly smaller subgroup by 39 ± 8% 6 months (*p* = 0.010) and by 35 ± 9% 12 months postoperatively (*p* = 0.013) compared to baseline. There was a significant effect of stimulation at both postoperative time points (F(1,4) = 7.40; *p* = 0.050) but no interaction effect between TIME*STIM. Improvement in chorea correlated with activation of the globus pallidus externus (GPe) 6 months after surgery on a trend level (*p* = 0.066; *r* = 0.82). After 12 months, the correlation of GPe activation with improvement of chorea was significant (*p* = 0.048; *r* = 0.90; Fig. 1).

**Fig. 1:**
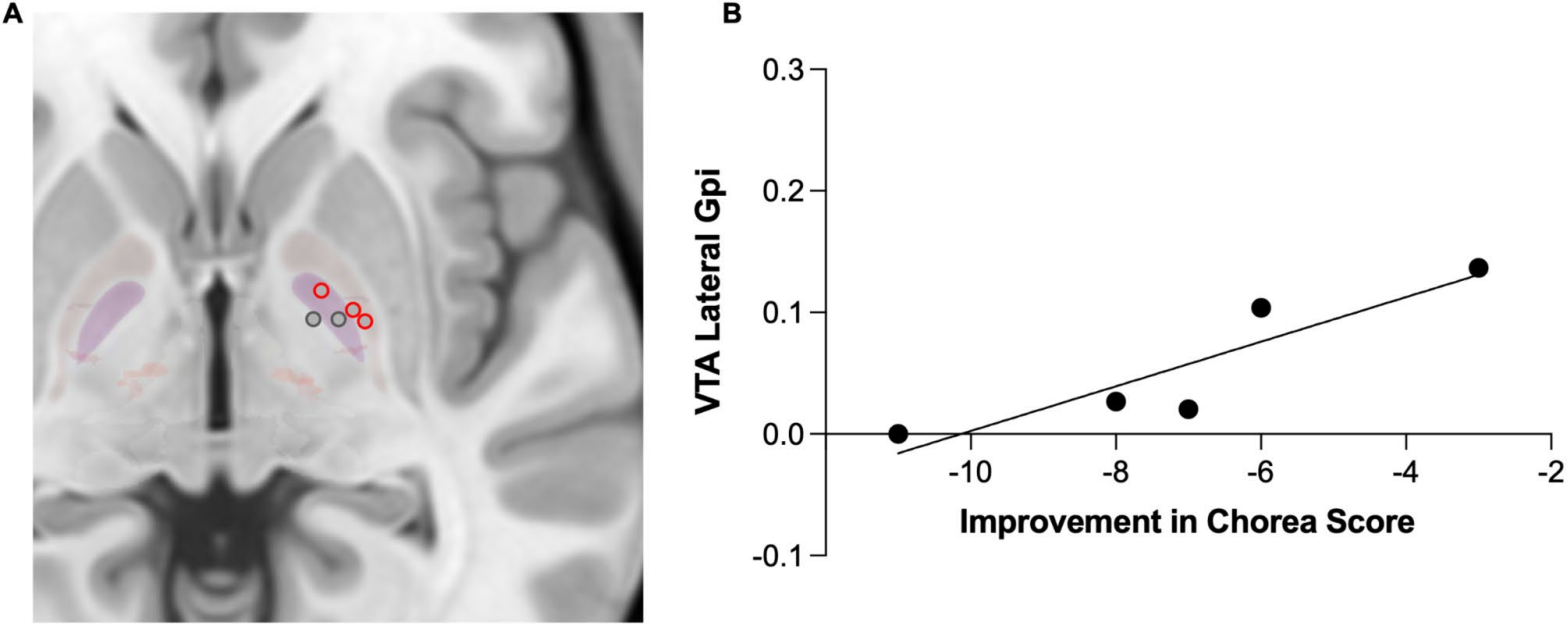
Target report of group electrode localization. (**A**) The leads and subcortical regions are shown in the TOR-PSM atlas. GPi/GPe border are highlighted in red. (**B**) Activation of the region lateral of the GPi correlated with improvement in chorea score 12 months postoperatively

Our findings suggest that the lateral GPi/GPe may be a potential DBS target in HD to alleviate chorea more effectively than classical GPi DBS as shown in previous work [6–10]. Segmented electrodes may help to estimate the expected individual benefit based on electrode positions and stimulation settings to provide a high degree of flexibility in the creation of a VTA. However, our study has certain limitations: brain atrophy may be associated with an altered basal ganglia microstructure making it difficult to differentiate between GPe and GPi. The number of patients is small and the patients were only evaluated up to 12 months postoperatively. Therefore, long-term effects of the surgical procedure are unknown. Our study should motivate to implant directional DBS leads when operating on patients with HD with the aim to improve chorea for a better clinical evaluation of stimulation effects in different subregions in the pallidal area.

## Data Availability

The datasets used and analysed during the current study are available from the corresponsing author on reasonable request.

## Abbreviations

DBS: Deep brain stimulation
GPe: Globus pallidus externus
GPi: Globus pallidus internus
VTA: Volume of tissue activated
